# The lung is a megakaryocyte outpost that can defend against thrombocytopenic attack

**DOI:** 10.1172/JCI186111

**Published:** 2024-11-15

**Authors:** Anthony K. Yeung, George J. Murphy

**Affiliations:** 1 Department of Pediatrics, UCSF, San Francisco, California, USA.; 2 Center for Regenerative Medicine of Boston University and Boston Medical Center, Boston, Massachusetts, USA.; 3 Section of Hematology and Medical Oncology, Boston University School of Medicine, Boston, Massachusetts, USA.

## Abstract

Lung megakaryocytes (Mks) are a unique subset of Mks that are distinct from their bone marrow counterparts. Recent evidence suggests that lung Mks favor an immune phenotype, but have unclear contributions to the total platelet mass. In this issue of the *JCI*, Livada et al. used an array of complementary in vivo labeling and tracing models in mice to investigate a longstanding question of where lung Mks are derived. By combining these models with stressed conditions, the authors assessed the contribution of lung Mks to total platelet counts in a homeostatic and thrombocytopenic state. Mks were minor contributors to the circulating pool of platelets during homeostasis but increased output during thrombocytopenia. These findings add critical understanding to the development of lung Mks and demonstrate the dynamic potential of these specialized cells to respond to thrombocytopenia.

## Adding a chapter to the lung megakaryocyte origin story

Megakaryocytes (Mks) are rare, delicate cells found in the bone marrow (BM), lung, and spleen that produce clot-inducing platelets. While the presence of lung Mks was established over a century ago, little was known about their contribution to the circulating pool of platelets or whether platelet function differed as a function of Mk tissue residence (1, 2). Technological advances in the last decade have allowed for a more fine-tuned approach to investigating Mks, which by inherent design are prone to adhering to other cells. This makes isolation and subsequent manipulation very challenging. The Livada et al. group and others (3–5) have previously demonstrated that lung Mks may function as antigen-presenting cells to drive the immune response. However, the developmental origins and functional role of lung Mks remained unknown.

One of the primary aims of Livada et al. in this issue of the *JCI* was to establish a better understanding of lung Mk ontogeny (6). The authors first established that a subset of lung Mks are truly resident. Using the cell-permeable dye CFSE for oropharyngeal (OP) labeling and intravenous biotin labeling, they demonstrate that approximately 10%–15% of lung Mks, but not Mks from the spleen or BM, remained labeled for one month. Furthermore, approximately 5% of lung Mks remained CFSE labeled at four months. Interestingly, a one-month time frame contrasts with the lifespan of BM Mks, which they found to only be about seven days. The authors also used a combined parabiosis and CFSE labeling model to demonstrate that approximately 80%–85% of lung Mks were replaced from the circulation throughout the month. The remainder stayed CFSE^+^ and did not intermix between parabionts. This finding suggests that lung-resident Mks, during homeostasis, do not leave the lung to reenter the circulation. How and when these resident Mks are mobilized into the circulation has yet to be determined. Others have demonstrated an increase in circulatory Mks in the setting of severe COVID infection and sepsis (7–9), suggesting the possibility that severe systemic inflammation or damage to lung parenchyma may mediate their release and functional activation. These circulating Mks may then contribute to mounting an appropriate immune response and/or the hypercoagulable state often seen in hyperinflammatory conditions such as sepsis or acute respiratory distress syndrome.

Livada et al. used the MDS1-Cre and FlkSwitch models to demonstrate that a large majority of lung Mks are hematopoietic stem cell (HSC) derived (approximately 85%) and differentiated from a Flt-3–independent lineage (85%–90%). In contrast, BM and splenic Mks are predominantly derived from a Flt-3–dependent lineage (10). These diverging lineages suggest there is a distinct mechanism by which Mks differentiate in the BM and are released into the circulation to populate the lung. Using a PF4-iDTR mouse model, the authors also assessed how this lung Mk population is maintained at both steady state and in response to platelet depletion. After depleting lung Mks, the authors showed that lung Mks were primarily reconstituted from the circulation and from local proliferation. Their work also suggested the possibility of differentiation from in situ local progenitors. Determining which mechanism dominates in response to a stressor or in murine models of disease is an important question for future studies (9). Such findings will optimize our capacity to harness the full therapeutic potential of Mks and their platelet progeny.

## Coming to a consensus on platelet census

**Figure 1:**
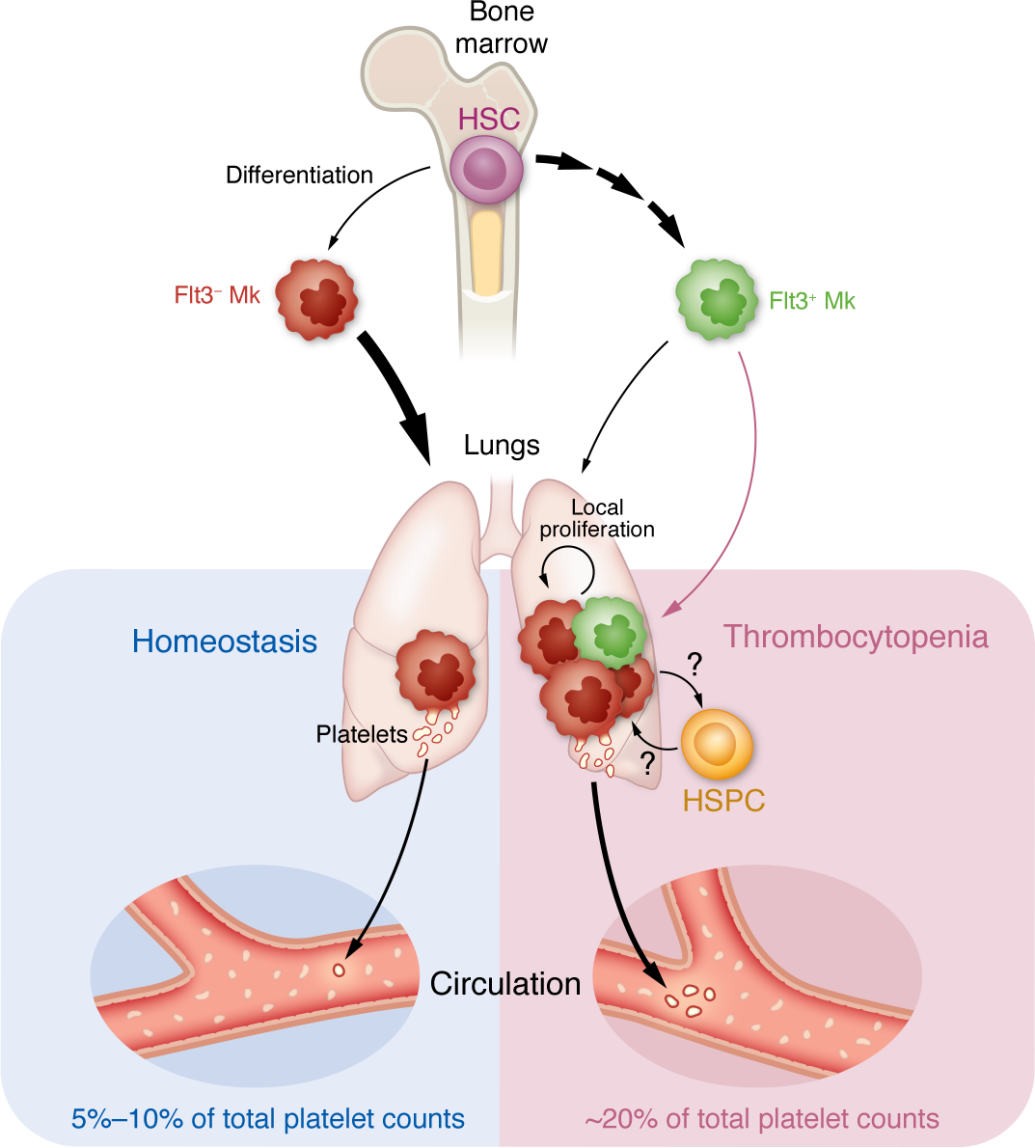
Livada et al. demonstrate that lung Mks contribute to the platelet pool. Under homeostatic conditions, lung Mks are preferentially derived from HSCs through an FLT3-independent pathway. Approximately 5%–10% of total platelet counts are derived from lung Mks at homeostasis. Models of acute thrombocytopenia via diphtheria toxin and chronic thrombocytopenia via malarial infection show that lung Mk thrombopoiesis contributes approximately 20% of total platelet counts. Increased platelet quantities coincided with increased Mk proliferation, migration of Fl3^+^ Mks from the bone marrow, and possibly differentiation from lung HSPCs. These lung-derived platelets have an increased responsiveness to TLR agonist and an increased rate of integration into platelet leukocyte aggregates.

The relative contribution of lung Mks contributing to the circulating platelet pool has been tackled by many investigators and remains a contentious topic. Previous estimates have ranged from 7%–98%, and limitations in the methodologies and calculations used to determine these estimates have been previously discussed elsewhere (1, 9). Livada et al. (6) delivered biotin and CFSE to the lung specifically to trace lung-derived platelets. After normalizing labeled platelet counts to labeled Mks, they concluded that approximately 5%–10% of total platelet counts were lung derived in steady state (Figure 1). There are, however, some notable drawbacks to this normalization.

To minimize cross-contamination, Livada and authors analyzed the lung Mk platelet contribution after an initial three- to five-day washout period. During this washout time, shorter-lived Mks might be lost, resulting in an underestimation of their contribution to the total platelet pool. This effect might be exacerbated if the longer-lived Mks that remain CFSE^+^ produce fewer platelets compared with those transiently present in the lung. Indeed, live, in vivo imaging of sessile lung Mks suggests that extravascular, quiescent Mks in the lung are smaller and produce fewer platelets (11), and others have suggested that thrombopoiesis is a carefully coordinated form of apoptosis that ultimately ends in Mk senescence (12–14). Therefore, while the authors’ approach advances our understanding of the contribution of lung platelets to the total circulating pool, more precise lineage-tracing studies are needed to accurately measure their relative contribution during homeostasis and disease.

## Harnessing the hematopoietic potential of the lung

Another notable finding from Livada et al. (6) was the disproportionate contribution of lung Mks to the circulating platelet pool during thrombocytopenia (Figure 1). Using both acute (PF4-iDTR with diphtheria toxin) and chronic (*Plasmodium yoelli* [PYnL], malaria) models of induced thrombocytopenia, the authors reported that lung Mks produced approximately 20% of the total circulating platelet pool during thrombocytopenia. This contribution was accompanied by a relative increase in overall lung Mk counts. The investigators also showed that infection with PYnL three weeks after CFSE labeling induced increased platelet production from long-lived Mks. Overall, this result suggests the lung has the capacity to augment platelet production in response to thrombocytopenia.

The authors also demonstrated that the platelets derived from long-lived Mks were more sensitive to TLR stimuli and integrated into platelet leukocyte aggregates at rates higher than CFSE^–^ platelets, suggesting a more immune-responsive role for lung-derived platelets. Further work is needed to determine how the function of lung-derived platelets differs from that of platelets in the BM in a disease context. Heterogeneity in platelet function should also be taken into consideration with in vitro–based platelet production, as tailoring platelet functionality to clinical needs will enhance patient outcomes. For example, maintaining competent platelet counts will help to minimize bleeding risks, but transfusing hypercoagulable platelets in the setting of sepsis may trigger disseminated intravascular coagulation.

The overall findings shared by Livada et al. (6) highlight the potential of the lung as a functional hematopoietic organ. Interestingly, the unique capacity of the lung microvasculature to support platelet biogenesis was recently demonstrated in an ex vivo mouse heart-lung model that served as a bioreactor to generate platelets (15). In addition to Mks, other groups have identified resident hematopoietic progenitor cells in the lung (11, 16). The juxtaposition of progenitors and Mks in the lung is of particular interest, given the symbiotic dynamics between hematopoietic stem-progenitor cells (HSPCs) and Mks in the BM. BM Mks are in close proximity to HSCs and can regulate HSC quiescence and proliferation (17–20). Meanwhile, HSCs have the capacity to differentiate directly into Mks (21, 22), a process that can be induced or modulated by stressors (23). Livada et al. (6) raise important questions with respect to the effects of Mk ontogeny on the production and function of circulating platelets. Their findings also raise questions about how the lung niche contributes to Mk, platelet, and HSPC function and how these cells communicate to coordinate the hematopoietic functions of the lung.

## Version 1 11/15/2024

Electronic publication
